# Handcrafted cuff manometers do not accurately measure endotracheal tube cuff pressure

**DOI:** 10.5935/0103-507X.20150037

**Published:** 2015

**Authors:** Raquel Annoni, Antonio Evanir de Almeida

**Affiliations:** 1 Department of Physiotherapy, Faculdade de Ciências Médicas de Pouso Alegre, Universidade do Vale do Sapucaí - Pouso Alegre (MG), Brazil.

**Keywords:** Intubation, intratracheal/instrumentation, Tracheostomy/methods, Transducers, pressure, Reproducibility of results

## Abstract

**Objective:**

To test the agreement between two handcrafted devices and a cuff-specific manometer.

**Methods:**

The agreement between two handcrafted devices adapted to measure tracheal tube cuff pressure and a cuff-specific manometer was tested on 79 subjects. The cuff pressure was measured with a commercial manometer and with two handcrafted devices (HD) assembled with aneroid sphygmomanometers (HD1 and HD2). The data were compared using Wilcoxon and Spearman tests, the intraclass correlation coefficient (ICC) and limit-of-agreement analysis.

**Results:**

Cuff pressures assessed with handcrafted devices were significantly different from commercial device measurements (pressures were higher when measured with HD1 and lower with HD2). The ICCs between the commercial device and HD1 and HD2 were excellent (ICC = 0.8 p < 0.001) and good (ICC = 0.66, p < 0.001), respectively. However, the Bland- Altman plots showed wide limits of agreement between HD1 and HD2 and the commercial device.

**Conclusion:**

The handcrafted manometers do not provide accurate cuff pressure measurements when compared to a cuff-specific device and should not be used to replace the commercial cuff manometers in mechanically ventilated patients.

## Abstract (pt)

**Objetivo:**

Testar a concordância entre dispositivos artesanais e um manômetro especificamente projetado para esse fim.

**Métodos:**

Testamos em 79 sujeitos a concordância entre as mensurações realizadas com 2 dispositivos artesanalmente adaptados para medir a pressão de balão dos tubos endotraqueais e as obtidas com uso de um manômetro específico para esse fim. A pressão de balão foi medida com um manômetro comercial e com dois dispositivos artesanais montados a partir de esfigmomanômetro aneroides. Os dados foram comparados utilizando os testes de Wilcoxon e Spearman, o coeficiente de correlação intraclasse e a análise do limite de concordância.

**Resultados:**

As pressões do balão medidas com os dispositivos artesanais foram significantemente diferentes das medidas obtidas com o dispositivo comercial (as pressões foram mais elevadas nas mensurações obtidas com o dispositivo artesanal 1 e mais baixas nas avaliações realizadas com o dispositivo artesanal 2). O coeficiente de correlação intraclasse entre o dispositivo comercial e os dispositivos 1 e 2 foi excelente (0,8; p < 0,001) e bom (0,66; p < 0,001), respectivamente. No entanto, os gráficos de Bland-Altman demonstraram limites amplos de concordância entre os dispositivos 1 e 2 e o dispositivo comercial.

**Conclusão:**

Os manômetros artesanais não proporcionam mensurações precisas da pressão do balão, quando comparados a um dispositivo específico para esse fim e, portanto, não devem ser utilizados na avaliação de pacientes mecanicamente ventilados em substituição aos manômetros comerciais específicos para avaliação da pressão do balão.

## INTRODUCTION

Tracheal tube cuff pressure is routinely assessed in subjects with tracheal tubes in intensive care units (ICU). The major goal is to prevent aspiration of the colonized secretions from the upper airways and mucosal injuries to the trachea.^(1,2)^ Hence, the authors recommend cuff pressures in the range of 20 to 30cmH_2_O;^(3-6)^ however, maintaining cuff pressures within these limits can be challenging in clinical practice.

The cuff pressure stability depends on several factors, such as compliance of the trachea and the cuff,^(7,8)^ the subject and cuff positions,^(9-11)^ the cuff volume^(6)^ and the body temperature.^(12)^ Because these factors vary continuously during an ICU stay, cuff pressure must be monitored and adjusted routinely.

A slight intracuff pressure excess, even for a short time, is sufficient to impair the local blood supply and cause hyperemia and hemorrhage in the tracheal wall at the cuff contact area.^(13)^ Castilho et al. analyzed the effect of using minimal cuff pressure to seal the airways of dogs (approximately 12cmH_2_O) for 60, 120 and 180 minutes. The authors showed that low cuff pressures were not able to prevent loss and disruption of tracheal epithelium, cilia loss, inflammation or blood cell infiltration in the cuff contact area.^(14)^ Nseir et al. also verified tracheal injuries juxtaposed to the cuff area, such as deep mucous ulceration, squamous metaplasia and intense mucosal inflammation, following 48 hours of intubation in piglets.^(13)^

Nevertheless, maintaining tracheal tube cuff pressure above 20cmH_2_O is fundamental to prevent the leakage of contaminated supraglottic secretions past the cuff. There are several factors related to ventilator-associated pneumonia (VAP), such as impaired host defense and mucociliary clearance, gastric and upper respiratory tract colonization and microorganism virulence;^(15,16)^ however, some authors affirm that the leakage of contaminated secretions past the cuff is the major etiologic factor for VAP.^(17,18)^ VAP is one of the most frequent infections in ICU patients,^(19)^ with a prevalence between 10 and 27%.^(20-22)^ Preventing the aspiration of respiratory secretions from the supraglottic space and monitoring cuff pressure are well-proven techniques to prevent VAP.^(23)^

Traditionally used in clinical practice, the cuff manometer is the recommended device for monitoring tracheal tube cuff pressures. However, due to its cost, some low-resource hospitals do not have the device. Alternative techniques and equipment have been explored to replace the cuff-specific manometer.^(24)^ As an economical option, Godoy and Vieira^(25)^ proposed a handcrafted device to measure cuff pressure, which is assembled with mercury sphygmomanometers, a three-way stopcock and a 5mL syringe.

These new devices have become popular among hospital staff due to their low cost and portability. However, with the declining use of mercury sphygmomanometers, handcrafted devices need to be produced with aneroid sphygmomanometers, such as the ones primary produced for measuring arterial blood pressure. Although it is believed that these devices are equivalent to the cuff-specific manometers because both are aneroid pressure gauges, their agreement has not yet been established.

The agreement between a new and a standard device should be tested before the new one can be used clinically. Agreement refers to how well readings from 2 different instruments agree. It is very unlikely that different instruments will agree perfectly when measuring exactly the same values. Nevertheless, if the limits of agreement (LOA) between the new and the standard devices are clinically acceptable, the devices can be considered interchangeable.^(26)^

Because handcrafted cuff manometers are widely used in clinical practice in Brazil, it is essential to ascertain their equivalence with a cuff-specific device. To our knowledge, there is no comparative study of these devices. Thus, our aim was to test the agreement between two handcrafted cuff manometers and a cuff-specific manometer in evaluating tracheal tube cuff pressures. Partial results of this study have been previously reported in the form of an abstract.^(27)^

## METHODS

This cross-sectional study was approved by the Institutional Ethics Committee of the *Universidade do Vale do Sapucaí*, Pouso Alegre, Brazil (1700/11). Informed consent was obtained from the subjects or their next of kin prior to the data collection.

A convenience sample of adult inpatients was prospectively studied between September 2011 and February 2012. All patients 18 years or older who were intubated with an oral tracheal or a tracheostomy tube for at least 24 hours were included. Subjects were recruited from the ICU or clinical wards of the *Hospital das Clínicas Samuel Libânio*, Pouso Alegre, Brazil. Participants were excluded if they met any of the following criteria: intubation for > 24 hours prior to the current hospitalization; head or neck surgery; a previous history of tracheal stenosis or tracheomalacia; a high risk of pulmonary aspiration; fever (> 38ºC); or positive end expiratory pressure > 12cmH_2_O. Demographic and clinical data were collected from the medical records.

**Figure 1:**
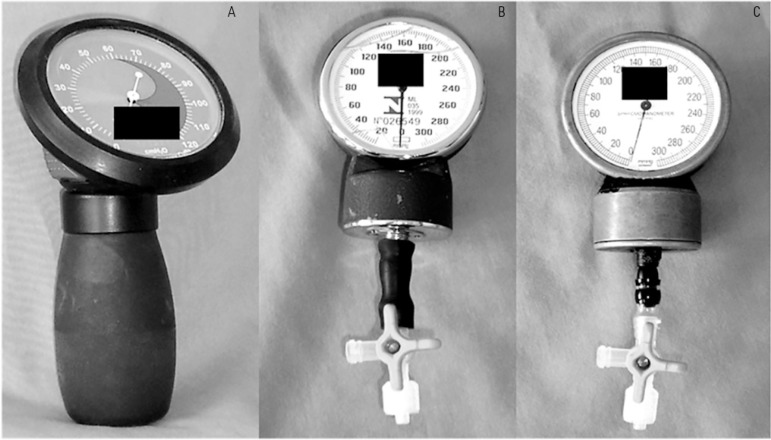
Commercial manometer (A); handcrafted devices 1 (B) and 2 (C).

Measurements of tracheal tube cuff pressure were obtained with three instruments: one cuff-specific and two handcrafted manometers. A handheld cuff manometer (JT Posey Company, Arcadia, California) was used as the standard technique (named as commercial device), as shown in figure 1. The assessment of cuff pressures was checked by attaching the commercial manometer extension to the tracheal tube pilot balloon via a three-way stopcock. The handcrafted devices (HD1 and HD2) were assembled with two aneroid manometers that had been removed from sphygmomanometers [HD1 (Solidor^®^, Lamedid, China); and HD2 (BD^®^, Sphygmomanometer, Germany)] and connected to a three-way stopcock.^(25)^ Cuff pressure was recorded by attaching the third limb of the stopcock to the cuff pilot balloon. All instruments were calibrated before the data collection.

For cuff pressure assessments, subjects were placed in supine position with the headrest at 30º. Three cuff pressure measurements were taken per patient, successively and randomly, with the three devices (one measure per device), by the same assessor. Cuff pressures were recorded at end-expiration and all data were collected during the 1:00 PM to 7:00 PM shift.

In the first month of the study, the measurements were obtained only with the commercial device and HD1. However, we observed that HD1 was inaccurate at pressures < 20mmHg because there was no gradation between 0 and 20mmHg. For this reason, another handcrafted device (HD2), which displays measurements in 2mmHg intervals, was included in the study.

For the measurements below 20mmHg assessed by HD1, the following values were considered: if the manometer pointer was exactly between 0 and 20mmHg, we registered 10mmHg. In the event that the pointer scored between 0 and what was considered 10mmHg, we recorded 5mmHg; and between 10 and 20mmHg, we recorded 15mmHg.

### Statistical analysis

The Kolmogorov-Smirnov test was used to test data distribution. As the data had a non-parametric distribution, Wilcoxon tests were used. Data are presented as median (IQR [range]) or the mean ± SD unless otherwise specified. The cuff pressures obtained with HD1 and HD2 were compared to the ones obtained by the commercial device. As the manometers showed different pressure units (mmHg and cmH_2_O), we converted the HD1 and HD2 values from mmHg to cmH_2_O (1mmHg = 1.36cmH_2_O). The correlation between the commercial device and HD1 and HD2 pressures was performed using Spearman’s coefficient.

To determine the degree of concordance between cuff pressures measured by two different instruments (commercial versus HD1 and commercial versus HD2), the intraclass correlation coefficient (ICC) with 95% confidence intervals (95%CI) was calculated. The ICC was interpreted according to Fleiss.^(28)^ Bland-Altman 95% LOA was used to evaluate agreement between the 2 devices (commercial device versus HD1 and commercial device versus HD2). The Fisher exact test was used to compare the characteristics of subjects who had their cuff pressures measured with the three devices and those evaluated with just HD1 and the commercial device.

The statistical power of the sample size showed 89% power (1-β, IC95%, two-tailed).^(29)^ A p-value of < 0.05 was considered significant. Statistical analysis was performed using Statistical Package for Social Science 15.0 software (SPSS Inc., Chicago, IL, USA) and GraphPad Prism 5 (GraphPad, San Diego, CA, USA).

## RESULTS

The subjects’ characteristics are presented in table 1. In total, 79 subjects [median (IQR) age of 53 (41 - 66) years, 65% male] were included in the study. Thirty-five [median (IQR) age of 54 (48 - 67) years, 66% male] had their cuff pressure evaluated just with HD1 and the commercial device, and 44 individuals [median (IQR) age of 52 (36 - 66) years, 64% male] were assessed with all three devices. Both groups had similar characteristics except for the type of tracheal tube used and the use of mechanical ventilation.

**Table 1:** Subject characteristics

	**Total**	Only HD1*	**HD1 + HD2** †	**p value** ‡
	**(N = 79)**	**(N = 35)**	**(N = 44)**	
Male	51 (65)	23 (66)	28 (64)	0.52
Age (years)	53 (41 - 66 [18 - 88])	54 (48 - 67 [24 - 82])	52 (36 - 66 [18 - 88])	0.49
OTT/TQT	74 (94)/5 (6)	30 (86)/5 (14)	44 (100)/0	0.01
Duration of tracheal tube use (days)	3 (2 - 5 [1 - 72])	3 (2 - 5 [1 - 72])	4 (2 - 6 [1 - 16])	0.74
Use of mechanical ventilation during data collection	74 (94)	30 (86)	44 (100)	0.01
Sedation during data collection	41(52)	19 (54)	22 (50)	0.44

HD - handcrafted devices; OTT - oral tracheal tube; TQT - tracheostomy tube. Values are number (proportion) or median (IQR [range]).

* This column corresponds to the subjects that had their cuff pressure measured just with HD1 and commercial device.

† This column corresponds to the subjects that had their cuff pressure measured with all devices (HD1, HD2 and commercial device).

‡ Comparison between subjects that had their cuff pressure measured with all devices and those evaluated just with HD1 and commercial device. Fisher exact tests were used.

**Figure 2:**
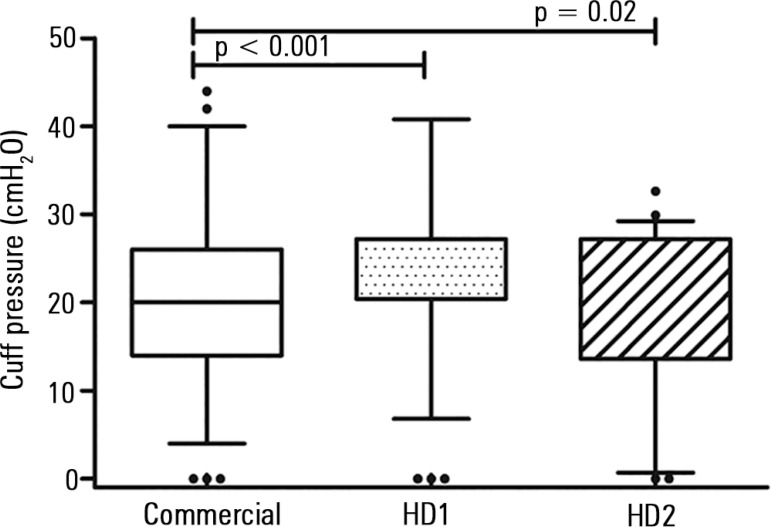
Tracheal tube cuff pressures (cmH_2_O) measured with a commercial manometer, handcrafted device (HD) 1 and HD2. Boxes indicate median and IQR range; whiskers indicate the 5 - 95 percentiles and dots indicate outliers. HD - handcrafted devices.

In comparison to the commercial device [median (IQR) 20 (14 - 26) cmH_2_O], cuff pressure values obtained with HD1 were higher [median (IQR) 20.4 (20.4 - 27.2) cmH_2_O, (p < 0.001)], whereas HD2 showed lower pressures [median (IQR) 13.6 (13.6 - 27.6) cmH_2_O, (p = 0.02)] (Figure 2).

**Figure 3:**
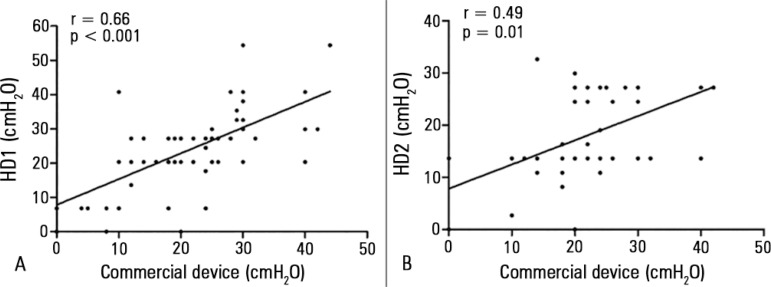
Correlation between cuff pressures (cmH_2_O) measured with commercial manometer and handcrafted device 1 (A) and 2 (B). HD - handcrafted devices.

A positive correlation was observed between the cuff pressures measured with the commercial device and HD1 and with HD2 (r = 0.66, p < 0.001 and r = 0.49, p = 0.01, respectively) (Figure 3). There was no correlation between cuff pressures and age or duration of tracheal tube use.

**Figure 4:**
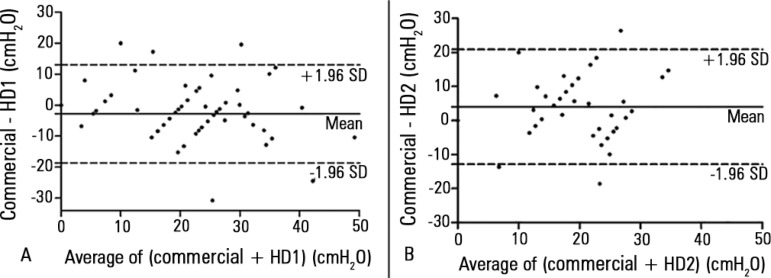
Bland-Altman plots showing the difference between cuff pressures from the commercial manometer and the handcrafted device 1 (A) and 2 (B), plotted against their mean, per subject. The mean difference is shown as the continuous line and the 95% level of agreement as the dashed lines. HD - handcrafted devices.

The ICC values indicated excellent concordance between the commercial device and HD1 [ICC = 0.8 (IC95% 0.68 - 0.87), p < 0.001] and good concordance with HD2 [ICC = 0.66 (IC95% 0.38 - 0.82), p < 0.001]. However, the Bland-Altman plots revealed a large mean (SD) difference between the commercial device and both HD1 and HD2 (-2.8 ± 8.1cmH_2_O and 4 ± 8.6cmH_2_O, respectively). In addition, there were wide 95% LOA values for HD1 (-18.6 to 13cmH_2_O) and HD2 (-12.8 to 20.9cmH_2_O) compared with the commercial device (Figure 4). Analyzing just the values between 20 and 30cmH_2_O (target cuff pressure), the mean (SD) difference and variability between the commercial device and HD1 and HD2 were -3.4 ± 7.5 and -18.1 to 11.3; and 3.6 ± 8.5 and -13.1 to 20.3, respectively.

## DISCUSSION

Measurements obtained with different instruments may be considered interchangeable when the mean difference between them is small and the variability is within acceptable limits.^(30)^ We demonstrated that tracheal tube cuff pressures measured with HD devices were higher (HD1) and lower (HD2) than those measured with the commercial device. Although the ICC has revealed excellent and good concordance among the manometers, large mean differences and variability were demonstrated by Bland-Altman plots. These results suggest that these handcrafted manometers cannot replace a commercial device.

To our knowledge, this is the first study that tested the agreement between two handcrafted manometers that were assembled with sphygmomanometers and a cuff-specific manometer. The replacement of an established instrument with another is possible only if the values measured by the new one are equivalent to those obtained by the established one.

The Association for the Advancement of Medical Instrumentation states that a sphygmomanometer may be replaced by another when the difference is less than 5mmHg and the variability is less than 8mmHg,^(31)^ which represents 5 - 10% of adult mean arterial blood pressure. To date, there is no recommendation regarding the difference and variability between cuff manometers. The commercial device used in this study has a specified accuracy of ± 2cmH_2_O, according to the manufacturer.^(32)^ As the mean differences with HD1 and HD2, observed in the Bland-Altman plots, were -2.8cmH_2_O and 4cmH_2_O, respectively, i.e., beyond the accuracy limits, replacement of the commercial device by the handcrafted instruments might not be safe.

Furthermore, although the concordance between the commercial device and HD1 and HD2 were excellent and good, respectively, from the ICC analysis, the variability observed in the Bland-Altman method should be taken into account when deciding whether to replace the commercial device with the handcrafted ones. Variations larger than 10% of the range recommended as safe represent a lack of reliability and might shadow hyperinflation or cuff deflation, leading to harmful complications. Bland and Altman argued that the smaller the variability, the better the agreement between two instruments.^(26)^ However, how small the range should be will depend on the clinical interpretation. If the variability between the two methods is clinically acceptable, they can be interchangeable.^(26,33)^ As the recommended cuff pressure range is very narrow (20 - 30cmH_2_O), we conclude that the variability should not be very large.

This study showed LOA between -18.6 to 13cmH_2_O for HD1 and -12.8 to 20.9cmH_2_O for HD2, compared to the commercial device. These results indicated a systematic variation (both up and down the bias) between the handcrafted and commercial instruments. Considering that the errors are more noticeable at higher cuff pressures,^(34,35)^ we analyzed the devices’ LOA using only the values between 20 and 30cmH_2_O. However, the systematic variation remained large.

Blanch evaluated the variability of 4 brands of cuff inflators and observed lower and upper 95% LOA from -2 to 3cmH_2_O, respectively. The study, however, compared only cuff-specific manometers and tests conducted on a trachea model, which can be limited compared with the human trachea.^(34)^

The variability of HD1 and HD2 may be explained not only by the handcrafted devices’ variation but also by the repeatability of the commercial device. In his study, Blanch tested the commercial device and observed that its variability (0.7 ± 1.9cm_2_O) trended both above and below the bias.^(34)^ Thus, the large LOA between the instruments shown in this study may be explained as a result of the sum of handcrafted and commercial device variability.

The dead space of manometers may also contribute to the systematic variation. Aneroid manometers contains volume even when they are not pressurized.^(34)^ As the pressure inside the cuff is higher than atmospheric pressure, by connecting the manometer to the cuff the pressures will be equalized, mainly due to cuff volume (and pressure) leak. Therefore, the accuracy of the cuff pressure measurement depends on the dead space size of each manometer and, consequently, on its volume and pressure before assessment.^(34,35)^ Because the manometers used in our study were produced by different manufacturers, it is possible that their dead spaces are different, influencing the variability between them.

Our study has some limitations. The HD1 was inaccurate for pressures below 20cmH_2_O. In addition, some authors state that up to 2cmH_2_O, or 1mL, are lost when the line is opened between the cuff and the pressure gauges.^(32,34,35)^ In our study, cuff pressures were evaluated using 3 devices sequentially, and air leakage over time may have contributed to the wide variability observed among the instruments. However, as we randomized the order in which the devices were used, the leakage over time was equally distributed among the instruments.

## CONCLUSION

In conclusion, handcrafted manometers do not provide accurate cuff pressure measures when compared to a cuff-specific device and should not be used to replace commercial cuff manometers in mechanically ventilated patients.
